# Human sporotrichosis: recommendations from the Brazilian Society of Dermatology for the clinical, diagnostic and therapeutic management^☆^

**DOI:** 10.1016/j.abd.2022.07.001

**Published:** 2022-09-22

**Authors:** Rosane Orofino-Costa, Dayvison Francis Saraiva Freitas, Andréa Reis Bernardes-Engemann, Anderson Messias Rodrigues, Carolina Talhari, Claudia Elise Ferraz, John Verrinder Veasey, Leonardo Quintella, Maria Silvia Laborne Alves de Sousa, Rodrigo Vettorato, Rodrigo de Almeida-Paes, Priscila Marques de Macedo

**Affiliations:** a Dermatology Department, University of Rio de Janeiro State, Rio de Janeiro, RJ, Brazil; b Department of Mycology, Brazilian Society of Dermatology, Rio de Janeiro, RJ, Brazil; c Evandro Chagas National Institute of Infectious Diseases, Fiocruz, Rio de Janeiro, Brazil; d Discipline of Cell Biology, Federal University of São Paulo, São Paulo, SP, Brazil; e Health School, University of Amazon State, Manaus, AM, Brazil; f Health Science Center, Federal University of Pernambuco, Recife, PE, Brazil; g Faculty of Medical Sciences, Santa Casa de Misericórdia, São Paulo, SP, Brazil; h Dermatology Clinic, Santa Casa de Misericórdia, Belo Horizonte, MG, Brazil; i Department of Dermatology, Santa Casa de Misericórdia, Porto Alegre, RS, Brazil

**Keywords:** Diagnosis, Laboratory, Recommendation, *Sporothrix*, Sporotrichosis, Treatment

## Abstract

**Background:**

The increase in the zoonotic epidemic of sporotrichosis caused by *Sporothrix brasiliensis*, which started in the late 1990s in Rio de Janeiro and is now found in almost all Brazilian states, has been equally advancing in neighboring countries of Brazil. Changes in the clinical-epidemiological profile, advances in the laboratory diagnosis of the disease, and therapeutic difficulties have been observed throughout these almost 25 years of the epidemic, although there is no national consensus. The last international guideline dates from 2007.

**Objectives:**

Update the clinical classification, diagnostic methods and recommendations on the therapeutic management of patients with sporotrichosis.

**Methods:**

Twelve experts in human sporotrichosis were selected from different Brazilian regions, and divided into three work groups: clinical, diagnosis and treatment. The bibliographic research was carried out on the EBSCOHost platform. Meetings took place via electronic mail and remote/face-to-face and hybrid settings, resulting in a questionnaire which pointed out 13 divergences, resolved based on the opinion of the majority of the participants.

**Results:**

The clinical classification and laboratory diagnosis were updated. Therapeutic recommendations were made for the different clinical forms.

**Conclusion:**

Publication of the first national recommendation, carried out by the Brazilian Society of Dermatology, aimed at the Brazilian scientific community, especially dermatologists, infectologists, pediatricians, family medicine personnel, and laboratory professionals who work in the management of human sporotrichosis.

## Introduction

Sporotrichosis, caused by species of the genus *Sporothrix*, is the most frequent subcutaneous mycosis in Latin America and Asia, although the first case was described by Schenck, in Baltimore, United States of America (USA).^1^ In Brazil, Lutz and Splendore were the first to report infections in rats and humans, and described the asteroid bodies in human sporotrichosis. Due to the epidemic of zoonotic transmission that started in Rio de Janeiro (RJ), the disease presented with unusual clinical manifestations, causing therapeutic difficulties that were not previously observed. The mycological diagnosis has been improved, especially the serology and there were contributions to molecular epidemiology and the phylogeny of *Sporothrix* spp. However, the last therapeutic recommendation for sporotrichosis was published 15 years ago, which is the reference used as the basis for this study.^2^

## Objectives

To update the classification of the clinical forms and laboratory diagnosis of sporotrichosis and recommend the most appropriate therapeutic choice for the new reality of this neglected mycosis.

## Methods

This study was conducted by the Department of Mycology of the Brazilian Society of Dermatology (SBD, *Sociedade Brasileira de Dermatologia*), 2021‒2022 term.

### Choice of components

Experts were defined as individuals with clinical and laboratory experience in caring for the population affected by sporotrichosis in different Brazilian regions, most of them from the southeastern region, where the epidemic started 25 years ago. Therefore, 12 professionals were invited to participate in the study and divided into three work groups: clinical, laboratory diagnosis, and treatment. Each group was led by the most representative participant in each category.

### Bibliographic search

The EBSCOHost platform was used with the following keywords: Sporotrichosis OR Sporothrix AND Diagnosis, Sporotrichosis OR Sporothrix AND Pathogeny, Sporotrichosis OR Sporothrix AND Treatment. The search included the English, Portuguese, Spanish and French medical literature, with no date limit, and resulted in approximately 2,800 articles. Few clinical trials were found, most of them open ones, with no randomized and controlled trials in human sporotrichosis, making it difficult to select articles based on robust scientific evidence. Review articles and those with new epidemiological, clinical-laboratory, or therapeutic knowledge were preferably selected. For the therapeutic recommendations, the ones with the largest number of cases were chosen.

### Division of topics, discussions and final consensus

The topics were discussed, written, and revised under the supervision of the leaders. The unified texts of the three groups were synthesized by the general coordinator in a draft, and later analyzed by all participants. Everyone participated in the discussions, by suggesting, disagreeing, suppressing, and finally approving the text through email, telephone contact, or virtual meetings, due to the COVID-19 pandemic. These discussions resulted in 13 disagreements, listed in a questionnaire, with three being related to pediatric treatment, five to local care and topical treatment, two to clinical aspects, two to immunoreactive forms, and one to writing. The answers were sent and returned in writing, individually, with the opinion of the majority prevailing. Finally, it was decided to list the recommendations on the therapeutic management of special situations faced in daily clinical practice.

After solving the disagreements related to the questionnaire, there was a final, remote hybrid meeting, and at the SBD headquarters, for the final approval of the text, tables, and figures. Any remaining questions or disagreements were solved after discussion.

## Results/discussion

### Epidemiology

Sporotrichosis affects both sexes and can occur at any age. Exposure to the fungus, either occupationally or recreationally, is a major factor in the disease development.^3^ Currently, the main areas of endemicity for sporotrichosis include Latin America, especially Brazil, Mexico, Colombia, Guatemala and Peru; Asia, especially China, India and Japan; and, to a lesser extent, the USA and Australia.^4^ As the disease notification is not always mandatory, the global epidemiological assessment is impaired. The incidence, reported in 2019 in the state of Rio de Janeiro (RJ), Brazil, was estimated at ten cases of sporotrichosis per 100,000 inhabitants.^5^

The importance of the domestic cat in the zoonotic transmission of sporotrichosis was first reported in the US in 1982 and later in São Paulo (SP), RJ and Rio Grande do Sul (RS), Brazil.^6^, ^7^, ^8^, ^9^ In recent years, northeastern states in Brazil, especially Pernambuco, Alagoas, and Rio Grande do Norte, have detected epizootic events among the feline population, with the consequent zoonotic transmission.^10^ By 2020, all Brazilian states, except Roraima, had published cases of human sporotrichosis.^11^

*Sporothrix brasiliensis* is the main agent of zoonotic transmission in Brazil and Argentina, although the zoonotic transmission of *Sporothrix schenckii* has been described in Brazil, the USA, Mexico, Panama, Argentina, India, and Malaysia.^12^, ^13^, ^14^, ^15^

Overall, the disease is benign; thus, data on mortality associated with sporotrichosis are scarce. In Brazil, 65 deaths have been related to sporotrichosis from 1992 to 2015.^16^

### Molecular epidemiology

Isolates from human and feline sporotrichosis cases in Brazil have shown the same genotypes, indicating that cats are the main source of *S. brasiliensis* infections.^17^ Phylogenetic analyses show that *S. brasiliensis* is a monophyletic species, with low haplotype diversity, low genetic recombination, and a small number of mutations, suggesting a clonal reproductive mode.^18^, ^19^ *Sporothrix schenckii* and *S. brasiliensis* must have diverged 2.2 to 3.1 million years ago, indicating that they already existed in a hitherto unknown habitat, even before the emergence of zoonotic sporotrichosis in these states.^20^

There is a great diversity of *S. brasiliensis* genotypes circulating in the Brazilian territory. However, epidemiological trends show that the recent geographic expansion of sporotrichosis transmitted by cats occurs through a founder effect, considering the low diversification of *S. brasiliensi* sisolates occurring in epizootic events and zoonoses. This pattern is supported by continuing founder events (e.g., constant migration of sick cats to new areas), leading to predominantly clonal outbreaks in a naïve host population. Additionally, molecular epidemiology studies indicate the state of Rio de Janeiro as the possible center of origin that led to the spread of the disease to other regions of Brazil, given the regular presence of the *S. brasiliensis* genotype from Rio de Janeiro in adjacent states such as São Paulo, Minas Gerais, and Espírito Santo, or even in areas further away from the epicenter, such as the state of Pernambuco.^21^

### Etiopathogenesis

*Sporothrix* spp. are found in living or decaying vegetation, animal excreta, and soil. The disease outbreaks are associated with the common source of transmission, as in the classic example of the South African gold mines, where the fungus was found on the timbers that supported the mines.^4^

Phylogenetic studies have shown morphological, physiological, genetic, epidemiological, and therapeutic differences between *Sporothrix* species.^22^ To date, 53 species have been recognized through phylogenetic analyses, including species from the clinical clade such as *S. brasiliensis*, *S. schenckii*, *S. globosa* and *S. luriei*.^11^

In most cases, the disease is caused by a single dominant molecular species: *S. brasiliensis* in southeastern South America (88%); *S. schenckii* in western South America, Central and North America (89%), in Australia and South Africa (94%); and *S. globosa* in Asia (99.3%).^23^ In Brazil, *S. brasiliensi*s, *S. schenckii* and *S. globosa* occur in sympatry. The first species is the most virulent in murine models and has been associated with atypical clinical features and severe forms of the disease, including systemic disease with cutaneous manifestation in immunocompetent hosts.^22^, ^24^, ^25^

All pathogenic *Sporothrix* species are thermodimorphic, appearing as filamentous fungi saprobiotically in soil, plants and animal excreta or *in vitro* at 25 °C, and as yeast-like structures in host tissue parasitism or *in vitro* at 35° to 37 °C.^9^

*Sporothrix* spp. are transmitted mainly by traumatic inoculation of material contaminated with fragments of hyphae or conidia into the skin or mucosa. Rarely, inhalation of fungal propagules and hematogenous spread, with or without cutaneous manifestation can occur, similar to what is seen in other systemic infections caused by dimorphic fungi.^9^ Classically, the environmental transmission of sporotrichosis is associated with soil manipulation activities, whether occupational or leisure ones. In the zoonotic transmission, the fungus is implanted in the skin from contact with animals, whether sick or not, that carry the fungus. The main animal involved in this process is the domestic cat, but other animals have been associated, such as parrots and other birds, squirrels and other rodents, fish, dogs, and insects.^9^, ^26^ In sick cats, the skin lesions contain a large number of parasitic fungal structures, thus showing a high zoonotic transmission potential.^27^ Laboratory accidents have been described with colonies of thermodimorphic fungi in the yeast-like form, in addition to the finding of yeast-like cells in the oral cavity of cats. The infection probably occurs due to the inoculum load associated with breaks in the skin, as in experimental animal infection.^28^, ^29^ The thermal tolerance of *S. brasiliensis* seems to be an important adaptation mechanism in these animals, whose average body temperature is 39 °C. This fact could explain, in part, why this animal is more commonly infected by this species. The identification of other forms of contagion is important; recently, two cases of fixed cutaneous (FC) and lymphocutaneous (LC) sporotrichosis on tattoos were described; the source of infection is suspected to have been the material used in the procedure, such as instruments, ink, or water.^30^

Presentation and clinical course of the mycosis depend on the amount and depth of the inoculum, the virulence of the pathogen, as well as the host immune response.^3^ After the implantation of *Sporothrix* spp. in the host, important changes occur in the fungus structure, including changes in temperature, pH and osmotic pressure, necessary for the adaptation to the new environment and, consequently, the transformation from the mycelial to the yeast-like phase. Some signaling pathways, such as heterotrimeric G-protein, Ras, and cAMP, as well as the mitogen-activated protein kinase (MAPK) cascade, seem to be important in inducing dimorphic transformation.^31^

The interaction of *Sporothrix* spp. cell wall with the host immune system triggers a mixed Th1/Th17 immune response, with the production of cytokines such as IFN-α, TNF-α and IL-17A, which activate macrophages and neutrophils. Macrophages can be activated by the production of IFN-β during the Th1 response, the most important cytokine in *Sporothrix* spp. infection. As for IL-17A, produced by Th17 cells, it is important in the repair and activation of epidermal barriers, and crucial for the control of natural killer cells. Another *Sporothrix* spp. mechanism to evade phagocytosis is ergosterol, a steroid present in the fungal membrane, which provides protection and prevents the destruction of the fungus by reactive oxygen species.^32^, ^33^, ^34^

The yeast-like cells of *S. schenckii* are capable of activating the complement system, both through the classical and the alternative pathways, with the latter being dependent on the presence of antibodies. Although the role of the humoral immune response against fungal pathogens has been better understood in recent years, little is known about the clinically relevant response against *Sporothrix* species. In a murine model, the humoral response seems to induce a partially protective immune response and control sporotrichosis.^35^

### Classification of the clinical forms

This updated classification facilitates pathophysiological understanding, diagnostic investigation, and patient management, particularly in light of changes in the clinical presentation of the mycosis in recent decades (Table 1).^9^

**Table 1:** Clinical classification of human sporotrichosis.

Clinical classification of human sporotrichosis^a^	
**Cutaneous**	Lymphocutaneous
	Fixed cutaneous
	Multiple inoculations
**Mucosal**	Ocular
	Nasal
	Others
**Osteoarticular**	Infectious arthritis/tenosynovitis/osteomyelitis – up to 2 foci^b^
**Systemic**	With cutaneous/mucous involvement
	With osteoarticular involvement
	Pulmonary
	Neurological
	Other locations/sepsis
**Immunoreactive**	Erythema nodosum
	Erythema multiforme
	Sweet Syndrome
	Reactive arthritis
**Mixed localized**^c^	Cutaneous + mucosa
	Cutaneous + osteoarticular
	Cutaneous + immunoreactive

a Modified from Orofino-Costa et al., 2017.

b By direct inoculation into the joint or by contiguity of the skin lesion.

c Without systemic involvement.

#### Cutaneous

**Figure 1:**
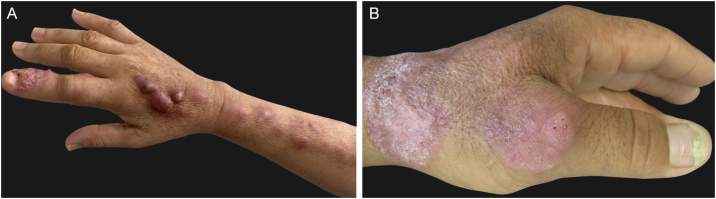
Clinical forms of human sporotrichosis. (A) Lymphocutaneous – inoculation chancre in the index finger and skin lesions in the ascending regional lymphatic pathway. (B) Fixed cutaneous form – verrucous lesion on the dorsum of the hand.

*Lymphocutaneous* (LC) ‒ It is the most common clinical form, accounting for 46% to 92% of cases.^36^ Clinically, it starts with the appearance, days to a few months after the trauma, of a small erythematous papule or pustule at the site of fungus inoculation. It is also called sporotichotic or inoculation chancre and usually is asymptomatic, tends to increase in size in a few weeks, and becomes nodular. Eventually, central liquefaction occurs, with fistulization or ulceration and subsequent drainage of purulent material (gummy lesion). After a few days to weeks, new papulonodular, erythematous, rosary-like lesions appear in the regional lymphatic pathway, which may be ascending or descending, depending on regional drainage (Fig. 1A). Similarly to the inoculation chancre, these lesions can become gummy and ulcerate. Although it occurs in any area of ​​the skin, places exposed to trauma, especially the upper and lower limbs, and the face, especially in children, are the most affected ones.^37^

*Fixed cutaneous* (FC) – It is the second most frequent clinical form, corresponding, on average, to 25% of cases. After the trauma, inoculation chancre appears, which does not show regional lymphatic progression, probably due to greater host resistance or a lower degree of thermotolerance and virulence of the fungal strain. The initial lesion may progress to ulceration, with irregular borders and varying sizes, or a verrucous appearance, with or without perforations for drainage of purulent material (Fig. 1B). The formation of a nodule is not rare; fluctuation and suppuration can occur, or it can be covered by scaly crusts. Sometimes small satellite papules around the initial lesion can be seen. In this form, the exposed sites of the body are also the most affected ones.^37^

*Multiple inoculation* ‒ It presents with multiple, polymorphic skin lesions in non-contiguous sites without systemic involvement. This clinical form has gained evidence with the emergence of zoonotic cases related to sick cats, in which the occurrence of multiple traumas is possible, due to scratches and bites. Overall, it affects immunocompetent individuals who usually report, consistently, the occurrence of multiple traumas; lesions appear almost simultaneously or in sequence.^9^ It is the least common cutaneous form.^37^, ^38^

#### Mucosal

**Figure 2:**
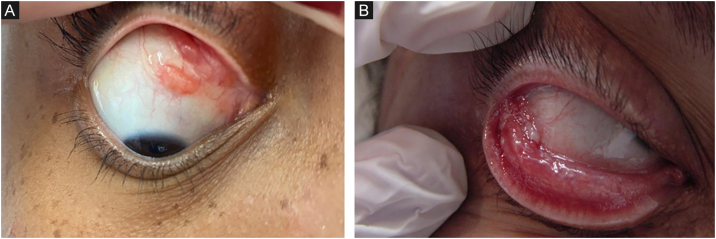
Clinical forms of human sporotrichosis. (A) Mucosal – bulbar conjunctiva lesion. (B) Mucosal – tarsal conjunctiva lesion, with pus.

Although any mucosa can be affected by *Sporothrix* spp., the ocular mucosa is the most commonly affected, due to greater exposure. The proximity between humans and domestic cats has increased the frequency of this clinical presentation, especially in children. When the animals sneeze, aerosols from the animals reach the human ocular mucosa, or, after touching the animal or fomites, the individuals bring the contaminated hands to the eyes.^37^, ^39^ The most characteristic clinical picture is granulomatous conjunctivitis, characterized by vegetating lesions on the palpebral and/or bulbar conjunctiva, and there may be enanthema and purulent discharge (Fig. 2A, B).^40^ It is possible for skin lesions to coexist on the eyelid ipsilateral to conjunctivitis. The presence of satellite lymphadenopathy, associated with ipsilateral granulomatous conjunctivitis, characterizes Parinaud's oculoglandular syndrome, often mistaken for cat-scratch disease.^41^ The involvement of the lacrimal sac can lead to dacryocystitis as a sequel.^42^ Episcleritis, uveitis, choroiditis, and other retrobulbar lesions rarely occur; these are often related to hematogenous spread in immunosuppressed patients, characterizing the systemic form with cutaneous/mucosal manifestation.^40^

The involvement of other types of mucosa is less common, with the nasal mucosa being the second most affected region. There are reports of lesions in the palate, pharynx and trachea.^43^

#### Osteoarticular

**Figure 3:**
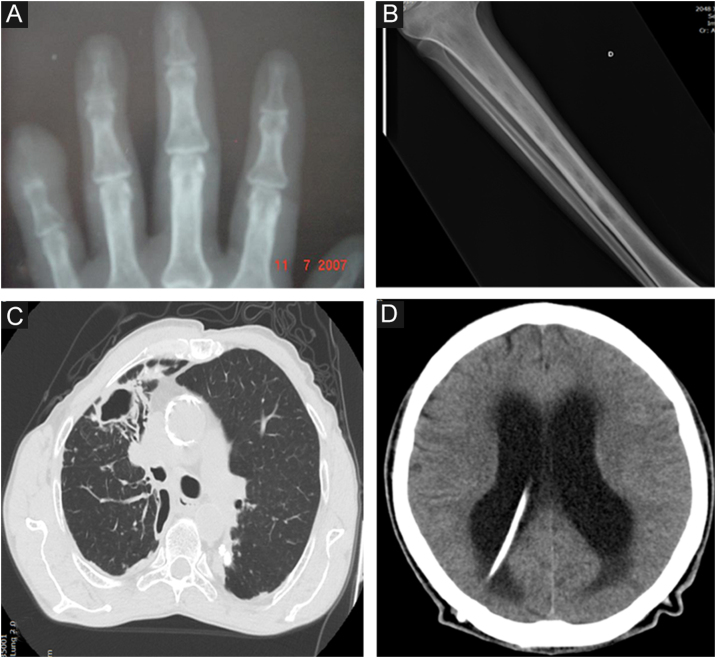
Radiological images in human sporotrichosis. (A) Osteoarticular form – resorption of the distal phalanx of the little finger caused by a cat bite (plain radiography). (B) Systemic form with osteoarticular manifestation – osteolytic lesions in the tibial medulla by hematogenous spread in a patient with systemic sporotrichosis and AIDS (plain radiography). (C) Pulmonary – cavity in the upper lobe of the right lung and extensive pulmonary opacity with a fibroretractile appearance (computed tomography). (D) Neurosporotrichosis – meningitis in a patient with systemic sporotrichosis and AIDS. Increase in the dimensions of the ventricular system, mainly in the supratentorial region (tetraventricular hydrocephalus), ventriculoperitoneal shunt catheter (computed tomography).

It is the most common clinical manifestation after the skin and the mucosa. The osteoarticular involvement usually occurs from the contiguous skin lesion, and is usually unifocal.^44^ In these cases, the most important risk factors are lesions in the extremities, especially hands and feet, due to the close anatomical proximity between the skin and the osteoarticular system and osteometabolic frailty, especially in the elderly or chronic users of corticosteroids; and bite wounds, due to the greater depth of the inoculum (Fig. 3A).

In unifocal osteoarticular lesions, joint involvement with cartilage destruction is observed, eventually showing synovial joint effusion, and osteolytic lesions, usually of the distal short bones of the hands and feet. It manifests with pain, swelling, and functional limitation. Other phlogistic signs such as flushing and heat are usually milder when compared to infections of bacterial etiology. *Sporothrix* spp. are frequently isolated from synovial fluid or fragments. Positive serology for *Sporothrix* ssp. has been demonstrated in synovial fluid.^44^

Osteoarticular involvement occurs in approximately 1% of the large series.^37^ In the largest of them, which included 41 cases, there was a predominance of multifocal bone involvement, associated with immunosuppression, especially AIDS, probably due to a selection bias by the profile of care in a referral center.^45^

Other manifestations are the involvement of the tendons, due to the contiguity of a skin lesion, characterized by tenosynovitis of the extensor and/or flexor tendons, particularly in the hands, regardless of bone and joint involvement. Imaging findings are characteristic and have been observed in zoonotic transmission, sometimes when the skin lesions have already healed.

To evaluate osteoarticular involvement in patients with extremity lesions, radiographic assessment is recommended, especially in cases of bite wounds associated with significant swelling or pain that is disproportionate to the skin condition. In these cases, bacterial osteomyelitis is considered a differential diagnosis due to the composition of the microbiota in the oral cavity of cats. Generally, in bacterial osteomyelitis, the findings are more acute, inflammatory, with a purulent exudate that has a foul odor, and are not always located as close to the entry point as in sporotrichosis. Ultrasound examination is indicated to evaluate tenosynovitis in cases with functional limitations that involve movements related to a specific tendon.^46^

#### Systemic

In the systemic form, which is rarer, other organs are affected, with or without skin lesions. It is accepted that, occasionally, the point of entry may be pulmonary with hematogenous spread of the pathogen, the traditional concept of 'systemic mycosis' proposed by Rippon, or it may show hematogenous spread from a cutaneous or osteoarticular lesion.^47^ Although there are no specific risk factors, patients with HIV/AIDS, with CD4+ counts < 200 cells/mm^3^, malnourished individuals, alcoholics, diabetics, transplant recipients, patients with hematological malignant neoplasias, in chronic use of immunosuppressive medications such as corticosteroids, which deplete cellular immunity, anti-TNF, and immunobiological, in addition to immunosenescence, are more predisposed to the development of systemic forms and may progress to death.^48^ Although *Sporothrix* spp. are primary pathogens, an opportunistic behavior of this mycosis can be observed in conditions of significant cellular immunity suppression, which may be an AIDS-defining disease.^42^ The systemic forms are rarely seen in immunocompetent individuals, which can be attributed to more virulent strains of the fungus or primary host immunodeficiency.^49^

*With skin/mucosal involvement* ‒ It must not be confused with primary cutaneous foci from multiple inocula or from primary mucosal inoculation. Hematogenous spread to the skin/mucosa is suspected when there are many skin lesions, dispersed in areas that are most often protected from trauma, such as the trunk, shoulders, proximal thighs, gluteal region, genitals, and face, especially the centrofacial region, or deep eye lesions, with reduced visual acuity, associated with a decline in the general status and underlying immunosuppression.^50^ Disseminated skin lesions may represent the only manifestation of hematogenous spread or be associated with the involvement of other organs, when they may be related to more severe conditions of immunosuppression, particularly HIV-induced cases.^51^, ^52^

They present with varied morphology, usually ulcerated, suppurative lesions, without odor or significant inflammatory reaction, which differentiates them from a secondary bacterial condition. Because they are suppurative, they are often covered by crusts, consisting of solidified pus and blood on the surface. Other types of skin lesions seen in these disseminated conditions are papules, nodules, verrucous plaques, necrotic, vegetative, tumor-like, and molluscum-like lesions.^52^, ^53^ Cold abscesses have been described in alcoholic patients.^50^

Mucosal involvement in systemic sporotrichosis, secondary to hematogenous spread, most often involves the nasal and oral (palate) mucosa. When the eye is affected by hematogenous spread, usually in immunosuppressed patients, posterior chamber involvement with chorioretinitis is observed.^54^, ^55^ Therefore, fundus examination is recommended in all patients with systemic sporotrichosis and clinical signs of hematogenous spread.

*With osteoarticular involvement* ‒ The involvement of the osteoarticular system through the hematogenous route is usually associated with invasive and intensely disseminated disease, particularly in AIDS patients, but it can also be seen in other immunosuppressive conditions.^45^ In these cases, there is multifocal involvement of long bones, with osteolytic lesions (Fig. 3B). Clinically, there is pain and functional limitation, and there may rarely be inflammatory signs, considering the low potential of inflammatory cell immune response in these patients.

Multifocal bone sporotrichosis is often oligosymptomatic, due to the depletion of cellular immunity. Systematic osteoarticular screening is essential, preferably by bone scintigraphy or, when this is not available, skeletal inventory with plain radiography of all long bones, in addition to the bones of the hands, wrists, feet, and ankles in patients at risk, especially those with AIDS.^45^

*Pulmonary* ‒ The involvement can be primary, through the inhalation of conidia and other infective propagules of *Sporothrix* spp., or secondary to hematogenous spread, usually from a primary cutaneous focus. When primary, it may be limited to the lung, as in chronic obstructive pulmonary disease (COPD), or spread from the lungs, which is common in immunosuppressed patients.^56^ In these cases, it is difficult to identify whether the dissemination occurred from that organ or from the skin. The history of trauma preceding the clinical picture can help to define the probable route of spread. It usually manifests as two clinical patterns:

Primary pulmonary ‒ patients have an underlying lung disease, usually smokers with COPD, with one or multiple cavitated lesions, associated with lung parenchyma fibrosis and architectural destruction (Fig. 3C).

Multifocal pulmonary ‒ it occurs in an immunosuppressed patient with sporotrichosis in other organs, in which the lesions are not normally cavitated.^56^ Immunosuppression due to chronic alcoholism and cavitated pulmonary lesions has been reported in a patient with disseminated sporotrichosis.^57^

Cavitated lesions confound the diagnosis of pulmonary tuberculosis, contributing to the underdiagnosis of pulmonary sporotrichosis, particularly in endemic regions.^57^ The main respiratory symptom is a persistent, dry, or productive cough for more than two weeks, similar to tuberculosis. The investigation includes a plain chest X-ray and CT scan, mycological examination, and sputum smear microscopy to exclude tuberculosis, mycobacteriosis, and other mycoses. Patients with AIDS may be oligosymptomatic.

*Neurological (neurosporotrichosis)* ‒ Involvement of the central nervous system (CNS) by *Sporothrix* spp. is a rare and severe condition. It usually occurs in more invasive pictures of the disease, particularly associated with AIDS, by hematogenous spread through the blood-brain barrier. In addition to host susceptibility, some *Sporothrix* species show greater virulence with neurotropism, especially *S. brasiliensis* strains. In neurosporotrichosis, subacute to chronic meningitis is usually present, although meningeal irritation may be mild or asymptomatic when there is significant depletion of cellular immunity (Fig. 3D). Early lumbar puncture to investigate CNS involvement should be routine in patients with AIDS and clinical signs of systemic sporotrichosis.^58^ On the other hand, neurological symptoms with more overt signs of meningismus may occur in immune reconstitution inflammatory syndrome (IRIS), which facilitates diagnostic suspicion.^59^ In neurosporotrichosis, the cerebrospinal fluid may be clear. There is an increase in cellularity at the expense of mononuclear cells, hyperproteinorrhachia, and hypoglycorrhachia. Due to the low parasitic load of *Sporothrix* spp. in the CNS, the isolation and identification of the fungus in culture samples is rare. In these cases, molecular methods seem to be promising for diagnostic investigation.

*Sepsis* ‒ It occurs when there is organ dysfunction due to generalized infection caused by *Sporothrix* spp. Although present in several organs, the isolation of the fungus from blood culture samples is uncommon. It constitutes the progression of other systemic forms in patients with a low capacity to react to infection, most commonly observed in association with AIDS. Any organ or system can be affected in sepsis caused by *Sporothrix* spp.

#### Immunoreactive

**Figure 4:**
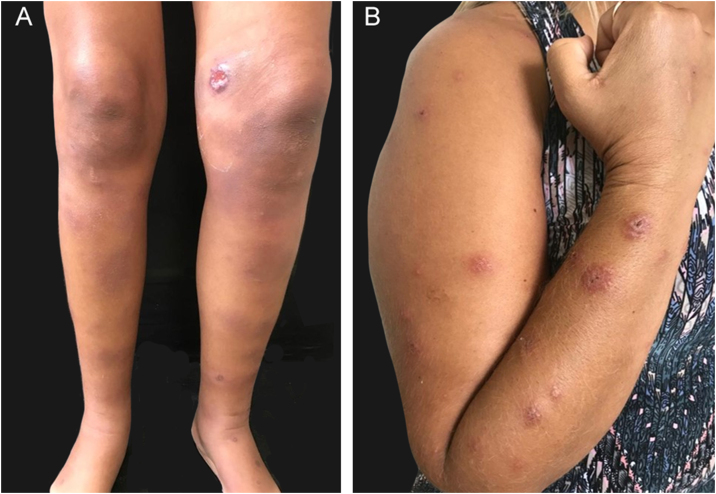
Immunoreactive forms of human sporotrichosis. (A) Erythema nodosum in the lower limbs (specific sporotrichosis lesion near the knee). (B) Sweet syndrome.

Patients with sporotrichosis cutaneous/mucosal lesions may develop hypersensitivity reactions in the course of the disease. These reaction forms have been seen most frequently in the zoonotic sporotrichosis epidemic, with erythema nodosum, erythema multiforme, and Sweet syndrome being the most common ones (Fig. 4A, B).^60^, ^61^ Reactive arthritis as a form of hypersensitivity, is overall polyarticular and migratory, disappearing before or after specific therapy.^62^ The immunoreactive forms are usually associated with milder and more localized sporotrichosis, which probably demonstrates better immunological control of the disease, although it is sometimes incapacitating. Exuberant reactive lesions may mask the specific lesions and the *forme-fruste* (subclinical form) of sporotrichosis.

#### Mixed

As in other diseases, there may be more than one form of clinical presentation in the same patient. For example, cutaneous and mucosal, cutaneous and osteoarticular, cutaneous and immunoreactive. One should not confound cases with mixed involvement of the skin, mucosa, and osteoarticular system with systemic sporotrichosis, whose involvement occurs by hematogenous spread.

#### Particularities in special groups

*Children* ‒ They have a higher risk of illness due to close contact with domestic cats and because they may present with atypical clinical conditions. The habit of petting domestic animals close to the facial region makes this topography prone to a greater risk of infection. Often, these atypical presentations delay the diagnosis, with a consequent higher risk of sequelae. On the other hand, children have a better immune response profile and cases are usually limited.

*Pregnant woman* – There is some difficulty related to the therapeutic management, as most pharmacological treatments are contraindicated.^63^ The possibility of fetal injury by the infection is also questioned.

*Elderly* ‒ Living with animals at home and the common comorbidities identified in this age group can lead to a greater risk of infection, potentially severe, and difficulty in therapeutic management, either because of the comorbidities themselves, such as diabetes mellitus or because of the drugs with which itraconazole, the first choice in the treatment of sporotrichosis, interacts. Osteometabolic frailty, which is typical of this age group, is also a risk factor for systemic and difficult-to-manage forms.^9^

*Immunosuppressed patients* ‒ Systemic sporotrichosis in patients with underlying immunosuppressive conditions, particularly HIV/AIDS, should be carefully evaluated by a multidisciplinary team, with the routine evaluation of multiple organs being recommended, and emphasis on nasal, oral and ocular mucosa (fundus examination), bones, joints, lungs and CNS.

### Dermoscopy

Dermoscopic findings in cutaneous sporotrichosis are not specific, as they overlap with those of other mycoses and leishmaniasis, depending on the morphology of the clinical lesion and its evolutionary stage. The most common are erythema, hemorrhagic crusts, yellow-orange areas, telangiectasias, and shiny white areas, which correspond to active lesions, the granulomatous phase, neoangiogenesis, and subsequent replacement by fibrous tissue.^64^ Therefore, to date, there is no consensus on specific dermoscopic aspects for sporotrichosis.

### Differential diagnosis

Infectious and non-infectious diseases, restricted to the skin or systemic, are included in the differential diagnosis (Table 2).

**Table 2:** Main differential diagnoses of clinical presentations in human sporotrichosis.

Cutaneous sporotrichosis		Mucosal sporotrichosis	Osteoarticular sporotrichosis	Systemic sporotrichosis
Lymphocutaneous	Fixed cutaneous			
American cutaneous leishmaniasis	American cutaneous leishmaniasis	Hordeolum/chalazion	Autoinflammatory disease	Tuberculosis
Pyodermitis	Pyodermitis	Bartonellosis	Arthritis or bacterial osteomyelitis	Histoplasmosis
Bartonellosis	Chromoblastomycosis	Foreign body granuloma	Trauma	Paracoccidioidomycosis
Atypical mycobacteriosis	Keratoacanthoma	Tuberculosis	Lyme disease	American cutaneous leishmaniasis
Tuberculosis	Squamous cell carcinoma	Sarcoidosis	Tuberculosis	Mucormycosis
Nocardiosis	Paracoccidioidomycosis	Histoplasmosis		Toxoplasmosis
	Venous/arterial ulcer	Entomoftoromycosis		Syphilis
	Tuberculosis verrucosa cutis	Paracoccidioidomycosis		Lymphoma
	Hyalohyphomycosis	American cutaneous leishmaniasis		Systemic hyalohyphomycosis
	Phaeohyphomycosis			Systemic phaeohyphomycosis
	Granuloma annulare			

### Laboratory diagnosis

#### Sampling methods

**Figure 5:**
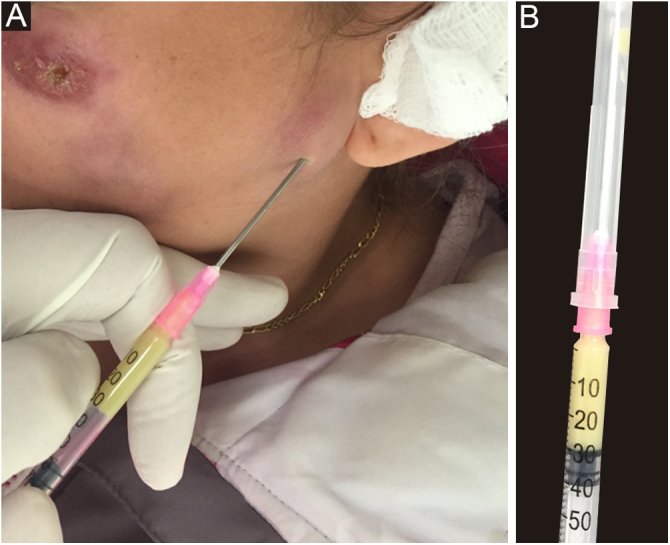
**Sampling.** (A) Purulent exudate, sampled by puncturing a skin abscess, in human sporotrichosis. (B) Material (pus) to be sent for mycological examination.

**Figure 6:**
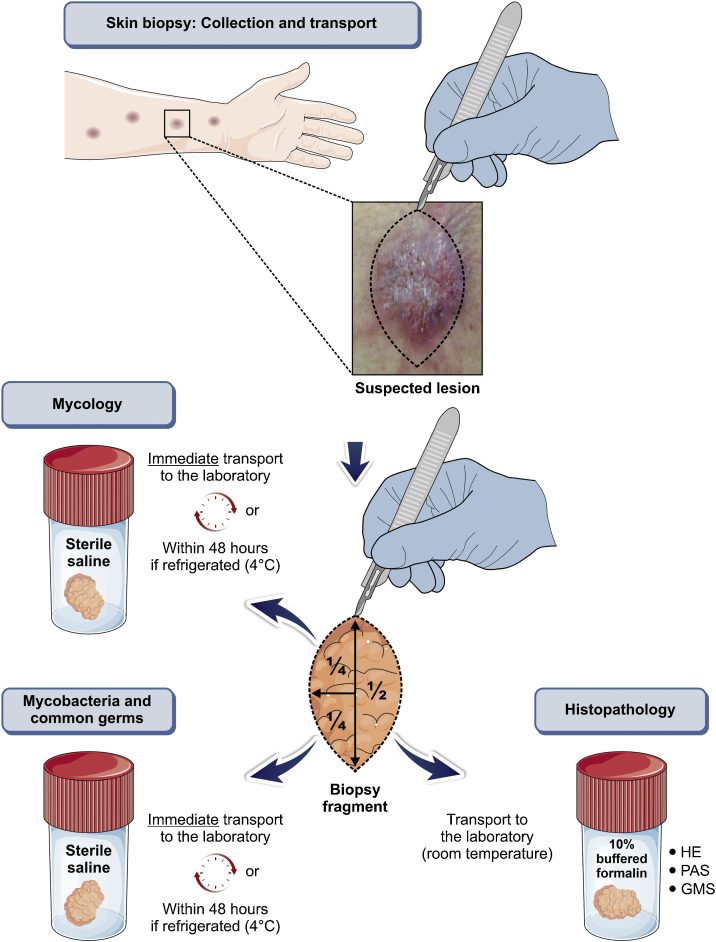
Flowchart for the collection and transportation of material sampled by biopsy, for the diagnosis of human sporotrichosis. The illustration was partially based on Servier Medical Art elements, licensed by Creative Commons Attribution 3.0 Unported License.

Any clinical specimen is suitable, although pus is the best material for the diagnosis.^9^, ^65^ It can be obtained by puncture of abscesses using a needle (Fig. 5) or by deep manual expression of lesions, especially after crust removal. These sampling methods are simple, fast, and cost-effective. If this is not possible, a biopsy of the lesion is the method of choice. For that purpose, one can collect material through a punch biopsy or use a scalpel to obtain a spindle-shaped biopsy. When in doubt about the diagnosis of sporotrichosis, the best technique is the spindle-shaped biopsy, as the histopathological examination will differentiate it from other diseases, whether infectious or not. Another advantage of the spindle-shaped biopsy is the correspondence between the samples that will be sent for culture for fungi and common microorganisms or mycobacteria and the histopathological examination. In both cases, the depth of the biopsy is essential for the correct diagnosis and it should extend to the subcutaneous tissue. Fig. 6 outlines the division of the sampled fragment and the transportation of the material to the respective laboratory.

#### Mycological examination

*Direct mycological examination* (DME) – It has low sensitivity and specificity in human sporotrichosis, especially in the LC and FC forms.

*Isolation* ‒ It is the reference method for the definitive diagnosis of sporotrichosis from clinical specimens in culture media. On Sabouraud dextrose agar and Mycosel agar media, *Sporothrix* spp. appear in 3 to 6 days, at 25° to 28 °C, in samples from skin lesions, and in 10 to 19 days from other organic materials. This may vary according to the species. ^66^

### Identification

**Figure 7:**
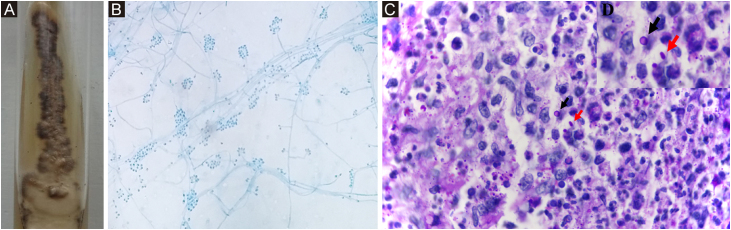
*Sporothrix* ssp. (A) Macromorphological aspect of the colony on Mycosel agar, at room temperature. It has a membranous surface, with a pearly luster, whitish color, surrounded by a blackened halo. (B) Colony micromorphology, at room temperature, shows delicate, branched, septate hyaline hyphae and oval or rounded conidia in a “daisy” arrangement, at the conidiophore tip. Cotton blue stain, ×100. (C) Histopathological skin section showing suppurative granuloma and parasitic fungus. Epithelioid cells on the left, neutrophils and pyocytes on the right, round yeast-like (black arrow) and elongated or navicular fungal cells (red arrow) PAS, ×1000. (D) Detail of fungal cells.

Phenotypic ‒ *Sporothrix* spp. are identified by their macro and micromorphological characteristics (Fig. 7A, B). Some species, such as *S. globosa*, are sensitive to temperatures above 35 °C. Laboratory demonstration of thermodymorphism in the colony, isolated from the suspected lesion, confirms the phenotypic identification of *Sporothrix* spp.^67^

Molecular ‒ DNA Sequencing – Reference method for molecular identification of medically relevant *Sporothrix* species, followed by phylogenetic analysis. To perform the phylogenetic analysis, it is necessary to include reference sequences, derived from strains used in the description of the species (type strains), available in public databases such as GenBank (Available at: https://www.ncbi.nlm.nih.gov/genbank/). The phylogenetic analysis of *Sporothrix* can include a single marker or a combination of protein-coding loci, aiming at increasing the resolution of molecular identification. The ITS region is described as an excellent “barcode” marker and supports the monophyly of the clinical clade.^68^ DNA sequencing allows the investigation of the genetic diversity and population structure of the fungus during outbreaks and epidemics, thus clarifying the transmission and expansion routes of emerging agents such as *S. brasiliensis*.

DNA Sequencing-Independent Methods – These are useful for rapid diagnosis. In general, molecular techniques capable of differentiating *S. brasiliensis*, *S. schenckii*, *S. globosa* and *S. luriei* employ DNA extracted from pure culture and include restriction fragment length polymorphism-polymerase chain reaction (RFLP-PCR; CAL-RFLP with HhaI enzyme), species-specific PCR, rolling-circle amplification (RCA), random amplified polymorphic DNA (RAPD, T3B), amplified fragment length polymorphism (AFLP), and qPCR.^21^, ^23^, ^69^ Each of these techniques has different applications, ranging from routine clinical laboratory diagnosis, and investigation of population structure, to the development of robust ecological studies aiming at detecting *Sporothrix* spp. in environmental samples.^11^

The quality and quantity of the isolated DNA, as well as the molecular target used, have a great impact on a successful identification. Extractions that do not involve any purification steps, for instance, can inhibit the polymerase chain reaction due to the presence of impurities in the extracted material. However, DNA extraction methods that employ commercial kits have been satisfactorily used and are effective in extracting and purifying DNA from clinical samples.

MALDI-ToF MS (Matrix-Assisted Laser Desorption/Ionization Time-of-Flight Mass Spectrometry and Time-of-Flight Analyzer) ‒ Potential tool for rapid and specific identification of *Sporothrix* spp. from colonies grown *in vitro*, with considerable savings of material and labor. The reliability and accuracy of MALDI-ToF MS in the identification of *Sporothrix* spp. is comparable to currently used molecular methods.^70^

#### Histopathological examination

The histopathological pattern is usually granulomatous, most often suppurative, but epithelioid granulomas not otherwise specified, tuberculoid, in palisade, foreign body, and sarcoid granulomas may also be seen in decreasing order of frequency. In most cases, lymphocytes and plasma cells complement the inflammatory infiltrate.^71^ Special histological staining techniques, such as PAS and Gomori-Grocott silver methenamine, are used when there is clinical suspicion and a compatible histopathological picture. *Sporothrix* spp. show considerable variation in shape and relative size; from round to elliptical, from 2 to more than 6 μm in their major axes, and navicular with approximately 3 × 10 μm (Fig. 7C, D). Narrow-based budding is relatively frequent, usually single, rarely double or multiple, sometimes elongated, and club-shaped. But they can be oblong, of uniform width, including at the base, not showing a club-like shape. Additionally, the occurrence of misaligned budding in relation to the major axis of the mother cell, considered characteristic of *Sporothrix* spp, is important.^72^ If there is an abundance of fungal elements, immunosuppression or other mycoses are suspected, especially histoplasmosis (fungal elements are smaller) or cryptococcosis (capsulated and slightly larger fungus).

Asteroid bodies are extracellular structures, most often found inside abscesses, and correspond to the deposit of immunoglobulins around a yeast-like fungal cell. It represents one of the manifestations of the Splendore-Höeppli phenomenon, and it is observed as hyaline, fibrillar, or club-shaped material, in a radial arrangement, eosinophilic in H&E and PAS-positive staining.^73^

Histopathological features of erythema nodosum and Sweet syndrome associated with sporotrichosis may be indistinguishable from those seen in lesions related to other causes.^74^ However, in erythema nodosum, significant lobular involvement was reported in one case. In Sweet syndrome, epithelioid differentiation of histiocytes has been observed, which may indicate the histiocytoid variant.^75^

#### Antifungal susceptibility test (AST)

Two standardized methods are used to test the susceptibility of *Sporothrix* species to antifungals: the Clinical and Laboratory Standards Institute (CLSI) which proposes the microdilution method using inoculum obtained from the filamentous form of *Sporothrix* spp., and the European Committee on Antifungal Susceptibility Testing (EUCAST).^76^ Fluconazole, flucytosine, and echinocandins do not inhibit the growth of *Sporothrix* spp. *in vitro*; therefore, their inclusion in the AST is unnecessary.^67^, ^76^ Some *Sporothrix* strains can be inhibited by voriconazole. Itraconazole, terbinafine, posaconazole, and amphotericin B have varied antifungal susceptibility profiles, which justifies their inclusion in all ASTs of human pathogenic *Sporothrix* species, given the possibility of a high minimum inhibitory concentration (MIC).^77^ The cutoff values ​​for clinical isolates of *S. brasiliensis* and *S. schenckii* according to the CLSI methodology are, respectively, amphotericin B, 4 and 4 μg/mL; itraconazole, 2 and 2 μg/mL; posaconazole, 2 and 2 μg/mL and voriconazole, 64 and 32 μg/mL. Additional cutoff values for *S. brasiliensis* include ketoconazole, 2 μg/mL and terbinafine, 0.12 μg/mL.^76^

Treatment-refractory cases are not necessarily associated with high MIC or the development of *in vitro* resistance during antifungal treatment. On the other hand, sporotrichosis caused by non-wild-type strains, that is, with high MIC values, tends to require longer treatment duration and higher doses of antifungals than those recommended in the literature, in addition to the possibility of the development of sequelae.^78^

#### Immunological tests

*Intradermal test (sporotrichin)* – It detects a delayed-type hypersensitivity reaction using crude antigen obtained from *S. schenckii* cultures.^79^ It allows a presumptive diagnosis and may indicate previous exposure to *Sporothrix* spp. or occur by cross-reaction with other fungi. It is used in epidemiological investigations in endemic areas, although it is only available in research centers. In disseminated disease, the test may be negative due to anergy.^80^, ^81^

*Serology* – It is an alternative tool in the laboratory diagnosis of sporotrichosis, useful for systemic and atypical forms, and as a diagnostic screening tool.^23^, ^44^ Enzyme immunoassays, mainly enzyme-linked immunosorbent assay (ELISA) and immunoblotting are more sensitive, with faster results.^82^ ELISA, which uses the SsCBF (*Sporothrix schenckii* Con A-Binding Fraction) antigenic fraction, has a specificity of 90% and a sensitivity of 80%.^82^, ^83^ It can be applied to the analysis of different biological samples, in addition to blood, such as synovial fluid and cerebrospinal fluid, resulting in efficient clinical-serological correlation and cure control.^44^, ^62^ The enzyme immunoassay with *S. brasiliensis* exoantigens, despite its simple methodology, showed variations in results.

The monitoring of antibody titers may indicate relapse or failure of the established treatment; however, it is not commercially available for use in humans.^83^

**Figure 8:**
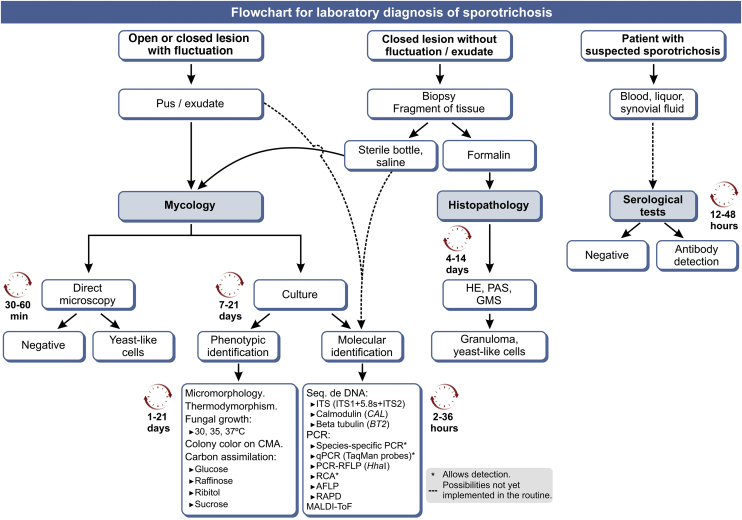
Flowchart for the laboratory diagnosis of human sporotrichosis, with estimated time for the processing of each method. GMS (Gomori-Grocott silver methenamine); CMA (cornmeal agar); 'C'‘C’, Carbon source; ITS, Internal Transcript Spacer; PCR, Polymerase Chain Reaction; qPCR, real-time quantitative PCR; RCA, Rolling Circle Amplification; AFLP, Amplified Fragment Length Polymorphism; RAPD, Random amplification of polymorphic DNA. Modified from Orofino-Costa et al., 2017.^9^

Fig. 8 shows an updated flowchart for the laboratory diagnosis of human sporotrichosis.

### Therapeutic recommendations

Table 3 shows the therapeutic recommendations for the main drugs, with the appropriate doses, as well as other therapeutic modalities used in the treatment of sporotrichosis, in its different clinical forms.

**Table 3:** Main therapeutic modalities used in human sporotrichosis.

Treatment	Clinical forms						
	Cutaneous (LC, FC, multiple inoculations) Mucosal		Osteoarticular^a^		Systemic^b^		Immunoreactive
	Immunocompetent	Immunosuppressed^1^	Immunocompetent	Immunosuppressed^1^	Immunocompetent	Immunosuppressed^1^	Hyperergic
Itraconazole^2^ 100 mg capsule	100–200 mg/d	200–400 mg/d	200–400 mg/d	400 mg/d	200–400 mg/d	400 mg/d	100 mg/d
Terbinafine^3^ 250 mg tablet	250–500 mg/d	250–1000 mg/d	Rarely	Rarely	Rarely	Not indicated as monotherapy	250 mg/d
Potassium iodide^4^ 0.07 g/drop aqueous solution	2.8‒3.5 g/d	Not indicated	Not indicated	Not indicated	Not indicated	Not indicated	2.8–3.5 g/d^5^
Amph. B deoxycholate^6^ 50 mg lyophilized powder	Rarely	0.5–1.0 mg/Kg/d	Rarely	Rarely	0.5–1.0 mg/Kg/d	0.5–1.0 mg/Kg/d	No indication
Amph. B lipid/liposomal complex^7^ 5 mg/mL suspension	Rarely	3.0–5.0 mg/Kg/d	Rarely	Rarely	3.0–5.0 mg/Kg/d	3.0–5.0 mg/Kg/d	No indication
Adjuvant^8^	Specific cases	Specific cases	Specific cases	Specific cases	Specific cases	Specific cases	Specific cases
Corticosteroid/ NSAIDs^9^	No	No	No	No	No	No	Yes

LC, Lymphocutaneous; FC, Fixed cutaneous; Amph. B, Amphotericin B; NSAIDs, Non-steroidal anti-inflammatory drugs.

a No systemic involvement, 6 to 12 months.

b All, including sepsis.

1 Dose depends on disease severity, long-term prophylactic antifungals may be needed.

2 1^st^ choice, as long as there is no contraindication; maintenance post-Amph. B for 12 months; liposoluble, to be administered with the main meal; can be administered as a pulse of 200 mg 2×/d/7 days/month; pediatric off-label use in moderate to severe cases, 3 to 5 mg/kg/d, maximum of 200 mg/d.

3 1^st^ choice in mild to moderate pediatric cases; can be used as post-Amph. B maintenance for 12 months if itraconazole is contraindicated; food does not interfere with the absorption; it can be administered as a pulse of 250 mg 2×/d/7 d/month.

4 1^st^ choice in mild pediatric cases, 1.4 to 2.1 g/day.

5 It can be used as monotherapy in hyperergic patients with mild symptoms; 1^st^ choice in immunoreactive cases, as long as there is no contraindication. Start with a lower dose in hyperergic patients.

6 Cumulative dose between 1 to 2 g or until clinical improvement.

7 2 to 6 weeks, until clinical improvement;^6,7^ do not use in saline solution.

8 Thermotherapy, cryosurgery, electrosurgery, excisional surgery, abscess drainage.

9 Associated with antifungal in symptomatic patients.

#### Medications

##### Potassium Iodide (KI)

KI was the first drug used in the treatment of sporotrichosis and remains a good therapeutic option, not only because of its low cost but also due to its rapid clinical response.^84^ It has an A-II level of scientific evidence and its safety and efficacy are well known.^2^ Its probable mechanisms of action are inhibition of granuloma formation through immunological and non-immunological mechanisms, action in neutrophil chemotaxis, *Sporothrix* spp. phagocytosis and biofilm inhibition in the yeast-like and filamentous phases.^85^, ^86^ The anti-inflammatory effect *in vivo* seems to be related to cytokine regulation, with increased levels of IL-10 and IL-35.^87^ It is likely that the anti-inflammatory and immunomodulatory effects are responsible for faster clinical improvement than with itraconazole or terbinafine.

Lower doses, administered twice daily, were used in an open-label clinical trial and showed the same efficacy and safety as those previously recommended in the literature (Table 4).^85^ The liquid form of the drug is an advantage for the administration to children and the elderly. It is useful in the treatment of the immunoreactive forms due to its anti-inflammatory effect, and as the first choice for children, in mild localized forms. The most common adverse events are headache, diarrhea, nausea, abdominal pain, and metallic taste, which rarely prevent further treatment. The coadministration of potassium-sparing diuretics and angiotensin-converting enzyme inhibitors increases the risk of toxicity (hyperkalemia). On the skin, acneiform rash and iododerm may occur. Transient reversible subclinical hypothyroidism is sometimes seen, which does not constitute the Wolff-Chaikoff effect.^84^ Investigating the patient’s personal or family history of thyroid disease and assessing thyroid function before treatment is recommended. It is contraindicated in renal failure, iodine allergy, autoimmune diseases, pregnancy and lactation, as well as in the disseminated and severe forms of sporotrichosis, as monotherapy. However, its successful use, in association with itraconazole in severe and refractory cases of feline sporotrichosis, may suggest that this association is a possible strategy for selected human cases.^88^

**Table 4:** Potassium iodide for the treatment of human sporotrichosis.^a^

	Daily dose in grams	Saturated solution 50 g KI (PA) in 35 mL of water (0.07 g/drop)	Concentrated solution 50 g KI (P A) in 50 mL of water (0.05 g/drop)
Adult	2.8–3.5 g/day	20–25 drops 2×/day	28–35 drops 2×/day
Pediatric	1.4–2.1 g/day	10–15 drops 2×/day	14–21 drops 2×/day

KI, Potassium Iodide; PA, Pure for Analysis; 1^st^ choice for children with mild and localized forms and for immunoreactive forms. Administer with milk or fruit juice. Start with five drops, saturated solution or seven drops, concentrated solution, 2×/day. Increase one drop per dose per day until the programmed dose is reached. Request blood count, biochemistry, TSH, T4L before and after one month of treatment.

a Adapted and modified from Orofino-Costa et al., 2013 and Macedo et al., 2014.

##### Azoles

*Itraconazole* ‒ It is a fungistatic triazole, which inhibits the synthesis of ergosterol from the fungal cell membrane and attains high concentrations in skin tissue.^89^ It has the level of evidence A-II, being the first therapeutic choice due to its safety and efficacy of 90% to 100%, in addition to the convenient dosage, provided there is no contraindication.^2^, ^89^ However, it is not the first choice for treatment in the pediatric population, since there are other effective therapeutic options and it is not included in the package insert due to a lack of research in this age group, although its off label use is justified in moderate to severe cases. Attention should be paid if handling it as a solution or syrup form, due to the liposolubility of itraconazole and the difficulty in measuring its bioavailability, pharmacodynamics, and, consequently, the actual dose being administered.

Headache, nausea, abdominal pain, diarrhea, or constipation are common adverse effects. As it is teratogenic and embryotoxic, it must not be administered to pregnant women, being considered category C.^9^ Its metabolism, dependent on CYP3A4, can cause serum variation of other drugs commonly used by the elderly or these drugs might influence the serum level of this azole.^90^ It decreases the serum level of oral contraceptives and is contraindicated in patients with hepatitis, severe dyslipidemia, and heart failure (negative inotropic effect on the heart muscle).^91^ Laboratory monitoring with blood count, biochemistry, lipid profile, and liver function tests is recommended before and 30 days after starting treatment. If the results are normal, testing should be repeated only after drug discontinuation.^9^

*Posaconazole* – It is a second-generation triazole, and has a good *in vitro* activity profile against *S. schenckii* and *S. brasiliensis*.^77^, ^92^ In Brazil, it is available as an oral liquid formulation, at a concentration of 40 mg/mL, and it is expensive. Doses of 600 to 800 mg/day have been used in severe cases with immunosuppression when there is intolerance to itraconazole and after intravenous therapy with amphotericin B, and further studies are needed to confirm the benefits, especially in *S. brasiliensis* infections. Although it has low penetration in the CNS, there are isolated reports of good clinical response in meningitis, possibly due to the increased permeability of the blood-brain barrier due to meningitis.^93^, ^94^

*Fluconazole* – It is not indicated for the treatment of sporotrichosis, except when other drugs cannot be used. Recurrence after drug discontinuation is common.

*Voriconazole* – Clinical studies are lacking; *in vitro* studies demonstrated little inhibition of *Sporothrix* spp. growth. 77, 92

*Ketoconazole* – Contraindicated in European countries, Australia, China and the USA; it is not recommended for sporotrichosis in Brazil.

##### Terbinafine

It is a fungicidal allylamine with an excellent concentration in fatty tissue, cornea, dermis, epidermis, and nails. It interferes with ergosterol synthesis by inhibiting fungal cell membrane squalene epoxidase, is metabolized by several CYP isoenzymes, has little interaction with other drugs, and is therefore useful in the elderly and in patients with comorbidities.^95^, ^96^, ^97^ It is risk category B for use in pregnancy, and penetrates breast milk; thus, the clinician should weigh the risk/benefit in these cases.^2^ It is the first-choice drug for mild to moderate cases in children over two years of age, with the doses recommended in the package insert (authors' experience). It can be used as an alternative if there is an absolute contraindication to itraconazole. Among the most common adverse events are headache, nausea, abdominal distention and pain, dyspepsia, and diarrhea.^97^ Caution is advised in patients with impaired liver function. It may precipitate or exacerbate pre-existing conditions of psoriasis and lupus erythematosus, probably mediated by exposure to ultraviolet radiation or by the influence of immunogenetic factors or characteristics.^9^

##### Amphotericin B

It is a polyene macrolide that adheres to the fungal membrane ergosterol, modifying its permeability. It is cardiotoxic and nephrotoxic, requiring the monitoring of kidney function and serum potassium levels. It is the only medication approved for use in pregnancy for the severe form of the disease, as it is not teratogenic.^63^

The Brazilian Ministry of Health provides the lipid complex for severe, disseminated, or unresponsive to oral treatment patients, through a systemic mycoses program, except for HIV/AIDS. Ideally, inpatient administration is preferred; however, in less severe cases, infusion on a day-hospital basis is possible, with daily applications or every two to three times a week.^98^ Sometimes the combination with itraconazole or terbinafine may be necessary.

#### Duration

It ranges from one to 12 months or longer, with a mean of 3 to 4 months in most published series.^2^, ^85^ Although some authors still recommend it, maintaining the treatment for 2 to 4 weeks after the lesions resolve is unnecessary. The identification of a clinical cure is crucial and is characterized by complete re-epithelialization, absence of exudation, crusts, infiltration, desquamation, or significant erythema. At this point, treatment can be discontinued without harm. Fibrosis, milia, hypertrophic scarring, mild erythema, pruritus, and local tenderness do not denote disease activity.^9^

#### New drug prospects

There are scarce data on the use of the new azoles in rescue therapy in cases that are refractory to conventional treatment.^99^ Isavuconazole, which has been approved in Brazil, has *in vitro* activity against *S. brasiliensis*, but there are few studies about it.^100^ Miltefosine, a phospholipid analog used to treat cutaneous and visceral leishmaniasis, has been tested *in vitro* and indicated as a possible therapeutic option, especially for patients who do not respond to conventional antifungals.^101^ Ongoing research evaluates whether herbal medicines and old drugs with other therapeutic indications can be useful for the treatment of human sporotrichosis, but still without practical perspectives.

#### Adjuvant treatment

Different modalities are used alone or in combination with systemic treatment and are useful for patients with intolerance or contraindication to systemic drugs, or poor response to therapy.^102^

*Thermotherapy* – It is most commonly used in pregnant women with uncomplicated clinical manifestations of sporotrichosis.^63^, ^103^, ^104^ It is based on the thermal intolerance of the *Sporothrix* species at temperatures above 39 °C.^3^ The heat source can be a hot water bottle, infrared source, or another method, aiming to reach a temperature of 42 to 43 °C for 20 to 30 minutes, three times a day.^105^ It promotes systemic drug permeation through vasodilation if used concomitantly.

*Cryosurgery* – Performed with liquid nitrogen in the proper equipment, with a non-contact spray tip, in two freeze/thaw cycles, monthly, until a clinical cure is achieved. The freezing time depends on the size and thickness of the lesion, approximately 10 to 30 seconds (Supplementary Material - video). The number of sessions depends on the clinical course. It is useful for the early treatment of verrucous and vegetating lesions. It is possible that penetration of systemic antifungals into the skin lesion may increase due to epidermal necrosis and consequent exposure of fungal antigens to the host immune system, shortening treatment time.^106^

*Electrosurgery* – for refractory cases, it is an exceptional resource, maintaining the systemic antifungal perioperatively and postoperatively to prevent dissemination.^107^ It is performed by a specialized professional and when associated with curettage, it constitutes an easy and simple method, maintaining local function and aesthetics. It can be indicated in places where cryosurgery offers a higher risk of complications, such as the nose and ears, for example.

*Others* – drainage or puncture of encysted/abscessed lesions and isolated curettage of verrucous-crusted lesions may help in the treatment of skin lesions by reducing the parasite pool, although there are no studies on these methods. There have been reports of successful use of photodynamic therapy, either alone or in combination with intermittent doses of itraconazole. ^108^

Recommendations in special situations:

1) Start treatment of the localized clinical forms found in immunocompetent patients with the lowest dose of each drug and wait at least one month for the clinical evolution. Rarely, larger doses of itraconazole or terbinafine are needed. If the patient does not progress well, it is better to associate other drugs or another therapeutic modality. Likewise, start with the lowest effective dose of KI. Higher initial doses increase toxicity and are no guarantee of rapid therapeutic response.
2) In classic cases of sporotrichosis with low therapeutic response, investigate the use of medications that reduce the absorption of itraconazole. If possible, measure the serum level of the drug, as it is an erratically absorbed medication. The use of proton-pump inhibitors is an example, as they reduce the absorption of itraconazole. Verify the concomitant medications on the itraconazole interactions list. Also, consider whether the antifungal was a compounding medication or if there was low adherence.
3) If there are drug interactions or contraindications to itraconazole, terbinafine or KI are effective treatment options. Both are well tolerated and have a low potential for interactions with other medications.
4) Give preference to terbinafine and KI for the treatment of children.
5) Be careful when medicating women of childbearing age, as systemic antifungals can decrease the concentration of oral contraceptives. The additional use of barrier contraceptive methods is advised.
6) Due to the teratogenic potential of azoles and the contraindication to the use of KI, it is advisable, during pregnancy and lactation, to use adjuvant physical methods for the treatment, with amphotericin B being reserved for severe cases only.^2^, ^102^, ^104^
7) In osteoarticular lesions, the initial dose of itraconazole should be 400 mg/day for six months.^2^, ^46^, ^109^, ^110^ After this period, it can be discontinued if cure is achieved, except in HIV/AIDS patients, when the drug should be maintained until CD4+ >200 cells/μL. If there are still signs or symptoms, the treatment continues with 200 to 400 mg/day for another six months (authors' personal experience). A synovectomy is eventually required.^2^, ^111^
8) There is still no consensus regarding the medication, dose and duration of treatment for pulmonary sporotrichosis, which should be guided by the clinical presentation severity. Mild cases can be treated with oral itraconazole and more severe cases with amphotericin B, followed by itraconazole. Most authors recommend a 6–12 month duration.^2^, ^56^, ^112^, ^113^
9) Surgical intervention is recommended for patients with localized lung involvement and/or those with radiological features of cavity disease.^56^, ^112^ Superior results are obtained with early surgery in combination with amphotericin B when compared to using drugs only.^56^, ^113^, ^114^
10) The administration of oral corticosteroids, such as prednisone, in symptomatic immunoreactive forms, plays an important role in neutralizing the exacerbated immune response, prescribed at a loading dose of 20 to 40 mg/day, or 0.5 mg/kg/day, providing it does not exceed 40 mg/day. Low doses, 20 mg/day, are usually sufficient. The corticosteroid withdrawal regimen is controversial, which can be carried out in 7 to 14 days, or by tapering off to prevent a recurrence.^63^, ^74^, ^75^ Erythema nodosum requires slower weaning. Mild and localized, oligosymptomatic cases can be treated with an antifungal associated with a non-steroidal anti-inflammatory drug (NSAID) or with KI alone, due to its immunomodulatory mechanism.
11) There may be a paradoxical response with exacerbation of the clinical picture, including the development of new lesions, at the beginning of the antifungal therapy. This phenomenon is due to the inflammatory process resulting from the release of antigens and the consequent immune response of the host. Treatment should preferably be performed with an antifungal agent associated with NSAIDs or potassium iodide alone, avoiding corticosteroids in these cases, due to the risk of worsening the infection or even the potential for invasion, especially of bones, in lesions located in the extremities. Reserve corticosteroids for moderate to severe immunoreactive forms, always associated with antifungal therapy. The emergence of new nodules in the lymphatic pathway may not mean worsening, but only a mechanism for the body to eliminate the disease.
12) The use of topical corticosteroids is contraindicated for sporotrichosis lesions, as they reduce local immunity and promote centrifugal growth or deepening of the lesion. Topical medications should be avoided because, in addition to having no effect, they can cause contact dermatitis. Washing the lesion with soap and water, without much friction, is sufficient, and the use of topical antiseptics is not necessary. Open lesions should be occluded to prevent myiasis, preferably by changing the dressing twice a day. Mineral oil or liquid petroleum jelly can be used with the dressing so that the gauze pad does not adhere to the lesion. If there are signs of secondary bacterial infection, systemic antibiotics should be preferred.
13) Controlling chronic diseases, reducing alcohol intake, and discontinuing steroid or anti-TNF use are important measures in all systemic forms of sporotrichosis, when possible.^48^
14) Try to reduce the dose of immunosuppressants and, if possible, discontinue the use of anti-TNFs in patients with autoimmune diseases. For that purpose, it is advisable to establish good communication between the medical teams involved in the treatment. Reducing the dose of immunosuppressants in transplant recipients may be the key to the successful treatment of sporotrichosis.^48^, ^100^
15) Serum levels of calcineurin inhibitors (tacrolimus and cyclosporine) increase significantly after treatment with itraconazole is initiated, so the monitoring of serum concentrations should be stringent.^100^
16) Amphotericin B is the main drug used to treat severe, visceral, and life-threatening systemic sporotrichosis, although the therapeutic response is not as favorable as in other systemic mycoses.^2^, ^48^, ^98^, ^115^ Treatment maintenance after the administration of amphotericin B is usually carried out with itraconazole for a period of 12 months.
17) Lipid formulations of amphotericin B constitute a more tolerable alternative to the conventional one; however, their superiority in terms of efficacy has not been proven for sporotrichosis.
18) Patients with HIV/AIDS seem to have a worse prognosis, requiring high doses of itraconazole, amphotericin B, and hospitalization due to lesion extension and/or comorbidities.^42^, ^98^, ^116^, ^117^
19) Beware of drug interactions between itraconazole and antiretroviral drugs such as efavirenz, ritonavir, and darunavir.^48^ The impact of implementing antiretroviral therapy (ART) during sporotrichosis is unknown and the best time for starting remains uncertain. Due to the predisposition to meningitis in IRIS, it is suggested to delay starting ART, similar to tuberculosis and cryptococcosis, in patients considered to be at high risk: neurological impairment, low CD4+ T-cell count, and high viral load.^118^
20) Lipid formulations of amphotericin B are the first choice in neurosporotrichosis, followed by maintenance with itraconazole for 12 months.^2^ Treatment withdrawal depends on the remission of the neurological signs and symptoms, and cerebrospinal fluid (CSF) cellularity should be monitored every 3 to 6 months until normal levels are reached (authors' experience).
21) The risk of recurrence of meningeal sporotrichosis is high in these patients; thus, it is prudent to maintain suppressive antifungal therapy or discontinue after a CD4+ T-cell count > 200 cells/μL in at least two separate measurements, with an interval of six months between them (authors’ experience).^118^ Generally, prolonged or even lifelong suppressive therapy with itraconazole at a dose of 200 mg/day is indicated when immunosuppression cannot be controlled, both in meningeal and disseminated involvement.^2^, ^49^, ^119^, ^120^

### Prognosis

The prognosis is usually good and cure is achieved, although it is slower in patients with immunosuppression and other comorbidities. The sequelae can range from the most frequent ones, such as hyper or hypopigmented, hypertrophic or keloid scars, to rarer ones, such as ankylosis or amputation of the extremities in cases of osteoarticular involvement, partial loss of tissue, such as auricular and nasal cartilage, or even perforation of the septum.^37^, ^45^, ^121^ Some patients will require reconstructive surgery for disease sequelae.

Cases of spontaneous regression were observed during the zoonotic epidemic.

Death can be an outcome in extreme cases of disease spread.^42^, ^117^ A poor prognosis is more frequent in individuals with HIV/AIDS and in people living in places where access to health services and socioeconomic conditions are poor.^115^

#### Prophylaxis

*Sporothrix* spp. are found in the environment; therefore, control and prophylaxis measures should be adopted, contemplating the concept of one health, especially in areas of higher prevalence, with a high concentration of people with little access to education, health, and sanitary conditions.^122^ These measures comprehend the use of adequate gloves, clothing, and footwear for handling plants and animals with cutaneous and/or mucosal lesions or when performing rural work, periodic cleaning of backyards, removal of construction material, and decomposing organic matter debris. Responsible care for animals should be encouraged, by treating the sick ones, and separating them from other animals and humans in the household, until the cure is achieved. Try neutering/spaying the cats, to avoid their going out for hunting, fighting, walking, or mating. Find viable means for the cremation of dead animals to prevent the fungus from perpetuating in nature. Actively offer information on the mode of transmission and prophylaxis of the disease to the owners of sick animals, aiming to encourage assisted treatment without abandonment.^3^ The abandonment of animals and the neglect of their care contribute to the perpetuation and increase of the epidemic.

## Conclusions

In this article, the authors updated the clinical presentations and diagnosis of sporotrichosis as well as therapeutic recommendations, in response to the new challenges presented by the zoonotic epidemic in Brazil.

## Financial support

None declared.

## Authors’ contributions

Rosane Orofino-Costa conceived and coordinated the project and participated in all stages until its completion. Dayvison Francis Saraiva Freitas, Priscila Marques de Macedo and Rodrigo de Almeida-Paes, led and coordinated the working groups and participated in all stages until the final approval.

Dayvison Francis Saraiva Freitas recorded the video, voiced by Rosane Orofino-Costa. Claudia Elise Ferraz, John Verrinder Veasey, Dayvison Francis Saraiva Freitas, Rosane Orofino-Costa, Priscila Marques de Macedo, Andréa Reis Bernardes-Engemann, Rodrigo de Almeida-Paes and Leonardo Quintella provided the clinical, laboratory and imaging photographs.

Andréa Reis Bernardes-Engemann was responsible for organizing the references and Anderson Messias Rodrigues for the schematic figures.

Rosane Orofino-Costa, Dayvison Francis Saraiva Freitas, Andréa Reis Bernardes-Engemann, Anderson Messias Rodrigues, Carolina Talhari, Claudia Elise Ferraz, John Verrinder Veasey, Leonardo Quintella, Maria Silvia Laborne Alves de Sousa, Rodrigo Vettorato, Rodrigo de Almeida-Paes and Priscila Marques de Macedo, participated in data collection, writing and reviews of the full text until its final approval. All authors read, agreed with and approved the final version.

## Conflicts of interest

None declared.
